# Impact of imlifidase treatment on immunoglobulins in an HLA-hypersensitized lupus nephritis patient with anti-SSA/SSB antibodies after kidney transplantation: A case report

**DOI:** 10.1016/j.jtauto.2023.100223

**Published:** 2023-12-01

**Authors:** Jean Milhès, Olivier Marion, Benedicte Puissant, Caroline Carlé, Charlène Bouthemy, Arnaud Del Bello, Nassim Kamar, Yves Renaudineau, Nicolas Congy-Jolivet

**Affiliations:** a Immunology Laboratory Department, Institut Fédératif de Biologie, Purpan, Toulouse University Hospital Center, Toulouse, France; b Nephrology and Organ Transplantation Department, Rangueil Toulouse University Hospital, Toulouse, France; c INSERM UMR 1291 - CNRS UMR 5051, Toulouse Institute for Infectious and Inflammatory Diseases (INFINITy), University Toulouse III, Toulouse, France; d UMR 1037 INSERM Team 20 / Université Toulouse III Paul Sabatier, Toulouse Cancerology Research Center (CRCT), Toulouse, France

**Keywords:** Systemic lupus erythematosus, Kidney allograft, Ides, Immunoglobulins, Donor specific antibodies, Anti-SSA/SSB antibodies

## Abstract

Bacterial recombinant cysteine protease Ides (imlifidase, Idefirix®, Hansa Biopharma) is used to prevent humoral transplant rejection in highly HLA-sensitized recipients, and to control IgG-mediated autoimmune diseases. We report the case of a 51 years old woman suffering from lupus nephritis with end stage kidney disease, grafted for the second time and pre-treated with imlifidase. The patient was HLA-hypersensitized (calculated Panel Reactive Antibodies [Abs], cPRA>99 %) and has three preformed Donor Specific Antibodies (DSA). Circulating immunoglobulins were monitored at initiation (0, 6, 36, 72 and 96 h), and at Ab recovery one and two months following imlifidase injection. From baseline, the higher depletion was reported after 36h for total IgG (−75 %) and IgG subclasses (−87 % for IgG1, IgG2 and IgG3, -78 % for IgG4), while no significant impact on IgA and IgM was observed. Anti-SSA 60 kDa and anti-SSB auto-Abs quickly decreased after imlifidase injection (−96 % for both after 36 h) as well as post-vaccinal specific IgG (−95 % for tetanus toxoid, −97 % for pneumococcus and −91 % for *Haemophilus influenzae* Abs after 36 h). At the Ab recovery phase, total IgG and anti-SSA60/SSB Abs reached their initial level at two months. Regarding alloreactive Abs, anti-HLA Abs including the three DSA showed a dramatic decrease after injection with 100 % depletion from baseline after 36 h as assessed by multiplex single bead antigen assay, leading to negative crossmatches using both lymphocytotoxicity (LCT) and flow cell techniques. DSA rebound at recovery was absent and remained under the positivity threshold (MFI = 1000) after 6 months. The findings from this case report are that imlifidase exerts an early depleting effect on all circulating IgG, while IgG recovery may depend in part from imlifidase's capacity to target memory B cells.

## Highlights

• Imlifidase is efficacious at initiation in removing alloreactive and autoreactive antibodies (Abs).
• Anti-SSA/SSB auto-Abs are restored two months after depletion.
• Donor specific Abs (DSA) recovery is lacking after 6 months.

## 1 Introduction

End stage kidney disease (ESKD) is reported in 10–20 % of patients with lupus nephritis (LN) [1,2], and kidney transplantation improves survival benefiting from reduction in infections, sepsis, and cardiovascular diseases as compared to dialysis [3]. Kidney injury leading to ESKD in LN results from multiple processes involving deposition of immune complexes (including anti-dsDNA and anti-SSA/SSB auto-antibodies [Abs]), thrombosis associated with anti-phospholipid Abs, complement activation, pro-fibrotic macrophage shift, and inflammatory reactions [4,5]. After kidney transplantation, LN-associated immunological injuries are limited with regards to long term transplant survival, although ESKD-LN patients are at risk for humoral acute rejection in the initial 12 months post-transplant [5]. In the context of hypersensitized patients with preformed donor specific Abs (DSA) awaiting a kidney allograft, which can be observed for ESKD-LN, removing alloreactive (allo)-Abs is a common therapeutic strategy to allow an HLA-noncompatible graft. According to the Eurotransplant Guideline for kidney transplantation, a patient is defined as highly sensitized when the percentage of panel reactive antibodies (cPRA) is higher than 85 %. For these patients, different therapeutic options exist to allow an allograft with DSA including the combination of plasma exchange, immunoadsorption, intravenous immunoglobulins (IVIg), anti-thymocyte polyclonal antibodies, and/or anti-CD20 monoclonal Ab [6].

In August 2020, the European Medicines Agency (EMA) approved a bacterial recombinant immunoglobulin G (IgG) serine protease derived from *Streptococcus pyogenes*: IdeS (GenBank accession number *ADF13949.1*, imlifidase: Idefirix®, Hansa Biopharma) to be used for desensitization treatment in highly sensitized adult kidney transplant candidates having a positive crossmatch assay against an ABO-compatible deceased donor [7]. The enzyme perfusion at 0.25 mg/kg quickly leads to IgG cleavage, generating single-cleaved IgG (scIgG) in the minutes following injection, then total cleavage is achieved in a few hours. This leads to a dramatic reduction of circulating total IgG including DSA, while F(ab’)_2_ and Fc fragments are still detected but unable to carry out complement dependent cytotoxicity (CDC) and Ab-dependent cellular cytotoxicity (ADCC) [8]. Imlifidase not only cleaves free IgG, but also the IgG-type of B cell receptor (BCR), affecting the memory B cell capacity to bind specific antigens and consequently their differentiation into Ab producing plasma cells [9]. In the phase II clinical study published by Jordan et al. [10,11], the use of imlifidase in highly sensitized patients benefiting from kidney transplantation leads after 6 h to anti-HLA Abs depletion, and after transplantation the graft survival rate reached 89 % with Ab-mediated rejection (ABMR) in 40 % of cases at 6 months. The use of IVIg (days 4 and 5) and rituximab (day 7) is delayed in the protocol in order to prevent any interference with the crossmatch result, and their introduction reduces anti-HLA Abs including DSA resynthesis [12]. To date, the longest follow-up of 39 crossmatch positive patients with imlifidase injection for kidney transplantation showed 84 % of allograft survival with 38 % of ABMR at three years [13]. These promising results has led to the use of imlifidase in other phase 2 studies in the ground of auto-immune diseases, including in Goodpasture syndrome. Patients with anti-glomerular basement membrane (anti-GBM) Abs that characterize Goodpasture syndrome develop acute kidney failure that can be prevented using imlifidase. Indeed, imlifidase injection led to depletion of anti-GBM Abs after 6 h, and a 6 month follow-up showed a better outcome compared to the standard of care for renal function in 67 % of treated patients [14].

In this case report, imlifidase was used to deplete DSA in a highly sensitized kidney transplantation candidate (cPRA >99 %) with ESKD-LN, allowing the study of quantitative and qualitative Ab depletion at initiation and next Ab rebound at recovery.

## 2 case report

Imlifidase was infused in a 51 years old woman with ESKD-LN in preparation for a second kidney allograft in the Toulouse University Hospital (January 2023). The medical history relates a patient diagnosed for LN at 24 years old, on the basis of a biopsy proven class IV proliferative LN, cutaneous injuries, and SLE associated Abs including anti-dsDNA and anti-SSA/SSB Abs. At the age of 39 years old she started peritoneal dialysis, and 3 years after, she benefited from a first kidney allograft from a deceased donor (May 2014). Two days after transplantation, vein thrombosis and iliac artery dissection led to a kidney transplantectomy. The patient was HLA-sensitized before the first kidney allograft, due to miscarriage, and the transplantectomy dramatically increased her sensitization. The patient became hyper-immunized, with anti-HLA allo-Abs directed against almost all HLA-A and HLA-B alleles except from herself (Thermofisher One Lambda® mean fluorescence intensity [MFI] for all HLA antigens between 2000 and 12,000; cPRA>99 %). She started peritoneal dialysis again and was then registered for the second time on the waiting list for kidney transplantation one year after the transplantectomy.

At the time of the second kidney allograft, there was no biological nor clinical activity of her LN (e.g., anti-dsDNA Abs undetectable) but the patient was still positive for anti-SSA 60 kDa and anti-SSB Abs (8.0 and 4.7AI, respectively, Bioplex, Biorad®). It was planned for her to undergo the IdeS protocol according to the criteria proposed by the French Consensus Guidelines [15]: (i) a kidney allograft with preformed DSA after imlifidase injection, (ii) cPRA>98 %, (iii) a deceased donor younger than 70 years old, with (iv) a modal kidney and (v) a negative lymphocytotoxicity (LCT) crossmatch on T and B cells 6 h after imlifidase injection and before transplantation. In our case, the allograft was performed with a 48 year-old deceased donor presenting good kidney function (creatinine: 36 μmol/L), a Pirche Matching Score of 224 [16], and a brief heart failure (0min of no flow). Three preformed DSA were present in the recipient: anti-A25 Abs, anti-B35 Abs and anti-B51 Abs (MFI 8900, 5700 and 8600, respectively, assessed by Luminex One Lambda® Single Antigen assay). Six hours (H6) after imlifidase injection, and before transplantation, LCT crossmatch was negative on T and B cells, which was compatible with a strong DSA MFI level reduction (see below). Given its better sensitivity as compared to LCT crossmatch, a confirmatory flow cell crossmatch on T and B cells was retrospectively performed confirming negativity at H6 as well as positivity before imlifidase injection (data not showed).

The allograft was performed according to the IdeS protocol [15]: 6 h before transplantation the patient received 0.25 mg/kg of imlifidase and 500 mg of methylprednisolone; then just before surgical graft starts, 1g of mycophenolic acid was added. After surgery, the immunosuppression protocol included 0.2 mg/kg/day of tacrolimus, 1g of mycophenolic acid the two days after transplantation, degressive doses (from 250 mg to 1 mg/kg) of methylprednisolone during the first week after allograft, 1.5 mg/kg of thymoglobulin from day 4 to day 8, 1 g/kg of IVIg at day 4 and day 5 and finally 375 mg/m^2^ of rituximab at day 7. The patient still presented good kidney function (creatinine = 87 μmol/L, creatinine clearance = 63 mL/min, hemoglobin 11.7 g/dL), and the kidney biopsy performed six months after transplantation excluded any humoral rejection. IVIg was infused three months after transplantation to treat a Parvovirus B19 active infection.

The following parameters were retrospectively explored: anti-HLA Abs by Single Antigen assay (One Lambda, Thermofisher®), IgG, IgA and IgM levels (Cobas, Roche®), IgG subclasses (IgG1-4) (Optilite, The Binding Site®), gamma-globulins electrophoresis (Capillarys, Sebia®), post-vaccinal Abs against tetanus toxoid, pneumococcus and *Heamophilus influenzae* (The Binding Site®) and auto-Abs against SSA 60 kDa and anti-SSB (Bioplex, Biorad®). Blood samples were collected at the following timepoints: just before imlifidase injection (H0), then at Ab depletion at H6 and H36, days (D)3 and D4, and at Ab rebound corresponding to months (M)1, M2 and M6 after injection.

**Fig. 1:**
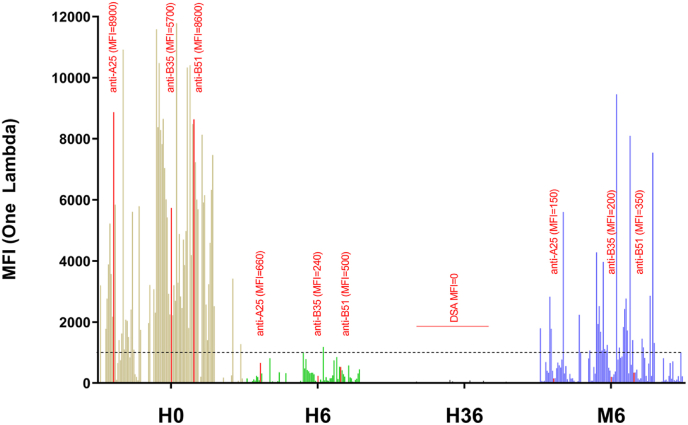
Imlifidase injection clears all anti-HLA antibodies (Abs). Anti-HLA Abs detected by the Single Antigen identification test (One Lambda MFI) have been represented for the following timepoints: hour (H)0 (in brown, just before imlifidase injection), H6 (in green), H36 (in black) and month (M)6 (in blue). Anti-HLA Abs are cleared at H6 (mean fluorescence intensity (MFI) < 100, dotted line) and most of them are undetectable at H36 (MFI = 0). The three donor specific antibodies (DSA) are presented for the four timepoints, and their MFI is indicated at each timepoint in red.

At treatment initiation corresponding to the Ab depletion phase, all anti-HLA Abs, including the three DSA, dramatically decreased at H6 (DSA MFI >90 % reduction compared to H0) leading to DSA negativity (anti-A25: MFI from 8900 at H0 to 660; anti-B35: MFI from 5700 at H0 to 240; anti-B51: MFI from 8600 at H0 to 500). At H36, anti-HLA Abs were undetectable (MFI<100 for all anti-HLA Abs) and, in particular, DSA (MFI: undetectable). Most of the anti-HLA Abs stayed undetectable until D5. Interestingly, we failed to observe DSA rebound at M6, for which MFI stayed under positivity threshold from H6 to M6 (anti-A25: MFI = 150; anti-B35: MFI = 200; anti-B51: MFI = 350 at M6) (Fig. 1).

**Fig. 2:**
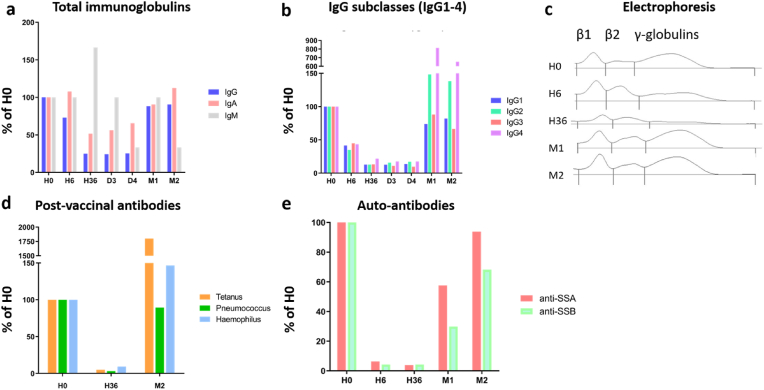
Kinetics of imlifidase depletion on total IgG, IgG subclasses, IgG post-vaccinal antibodies (Abs) and IgG anti-SSA 60 kDa and anti-SSB Abs at depletion phase (first week) and after after Ab rebound. Timepoints selected: hour (H)0 (just before imlifidase injection), H6, H36, day (D)3, D4, months (M)1 and M2. **A:** Imlifidase selectively depletes total IgG. without affecting IgM and IgA. An IgG recovery is observed at M1 and M2. **B:** Imlifidase effects on the four IgG subtypes IgG1-4. **C:** Imlifidase effects on gamma-globulins. Gamma-globulins are 57 % of H0 at H6, and 15 % at H36. **D:** Imlifidase effects on IgG post-vaccinal Abs against tetanus toxoid, pneumococcus and *Haemophilus influenzae*. **E:** Imlifidase effects on IgG auto-antibodies targeting SSA 60 kDa and anti-SSB Abs. After two months IgG anti-SSA and anti-SSB Abs are restored near to their initial level (93 % and 68 % respectively from H0).

As expected at the Ab depletion phase, IgG dramatically decreased following imlifidase injection, whereas total IgA and IgM were not affected by imlifidase injection. Total IgG started to decrease just after imlifidase injection, and reached 75 % of H0 at H6, and 25 % of H0 at H36, D3 and D4. The lowest concentrations were observed at D4 (2.33 g/L *versus* 9.63 g/L at H0) (Fig. 2a). All IgG subclasses showed a similar kinetic, with 40 % of H0 at H6 and 10–20 % of H0 at H36, D3 and D4. The lowest concentrations were obtained at H36 for IgG2 (0.24 g/L *versus* 1.93 g/L at H0), D3 for IgG1 and IgG4 (0.77 and 0.004 g/L respectively, *versus* 6.12 g/L and 0.023 g/L at H0) and D4 for IgG3 (0.02 g/L *versus* 0.22 g/L at H0) (Fig. 2b). All these parameters returned back to their initial level at M1 and M2 but Ab recovery partially reflects endogenous synthesis, as IVIg supplementation used at days 4 and 5 brought exogenous IgG. Electrophoresis drawings also reflected the IgG depletion through the progressive flattening of the γ-globulins area. γ-globulins concentration was 12.6 g/L at H0, 7.6 g/L (60 % of H0) at H6 and 2.7 g/L (21 % of H0) at H36 (Fig. 2c).

Post-vaccinal Abs also showed a dramatic decrease following imlifidase introduction. At baseline, IgG Abs against tetanus toxoid (0.02 mg/L) were present at low protection level (LPL = 0.01–0.10 mg/L), IgG Abs against *Haemophilus influenzae* (0.75 mg/L) at intermediate protection level (IPL = 0.15–1.0 mg/L), and IgG Ab against pneumococcus (48.4 mg/L) at IPL (10–270 mg/L). All of them decreased under the protective threshold at H36: 0.001 mg/L *versus* 0.01 mg/L (anti-tetanus toxoid Abs), 0.07 mg/L *versus* 0.10 mg/L (anti-*Haemophilus influenzae* Abs), and 1.57 mg/L *versus* 3.3 mg/L (anti-pneumococcus Abs). Taken together, at H36 more than 90 % of the three post-vaccinal Abs were degraded (Fig. 2d). As for total IgG, the recovery of the post vaccinal immunity at M2 cannot be fully attributed to endogenous synthesis, as part of the post-vaccinal Abs came from IVIg supplementation.

A similar kinetic has been observed for auto-Abs: 94 % of anti-SSA 60 kDa Abs and 96 % of anti-SSB Abs have been degraded at H6. The lowest values were obtained at H36 (0.3AI, and 0.2AI respectively, 4 % of H0 for both). Anti-SSA 60 kDa Abs and anti-SSB Abs progressively reached their initial level: 4.6AI (58 %) at M1 then 7.5AI (94 %) at M2 for anti-SSA 60 kDa Abs; 1.4 (30 %) at M1 then 3.2AI (68 %) at M2 for anti-SSB Abs (Fig. 2e). For note, anti-SSA/SSB Abs recovery could not be ascribed to IVIg administration, and then considered as endogenous IgG synthesis.

## 3 Discussion

Imlifidase efficiently cleaves all IgG independently of their specificity, their pathogenic role, their protective role, and their subclass. The resulting effect at the Ab depletion phase is a dramatic reduction in circulating IgG that is quickly obtained (H6), and maintained until D4. Importantly and as reported in our case report and by others [8,17], Abs can be detected at low levels by single antigen bead assay, ELISA, turbidimetry, and Western blot, but the enzymatic degradation leaves scIgG unable to correctly activate complement, explaining the negativity of the LCT and flow cell crossmatch assays at H6, although scIgG are detected. The presence of scIgG may also explain the dichotomy reported between total IgG and the sum of IgG subclasses retrieved at the initiation phase [17,18].

We also observed an important difference in the Abs kinetic between allo- and auto-reactive Abs at the recovery/rebound phase. Due to the use of IVIg at D4 and D5, Ab recovery of total IgG and post-vaccinal Abs kinetics need to be cautiously analyzed. However, such bias may not be ascribed to anti-HLA allo-Abs, nor auto-Abs (anti-SSA/SSB) which are negligible in IVIg preparations. Important differences were reported in our study as alloreactive DSA remained below the positivity threshold at M6, while the expected IgG recovery was reported for anti-SSA/SSB Abs at M1 and even more at M2. The mechanism of imlifidase on circulating IgG and of imlifidase coupled with immunosuppressors on memory B cells expressing IgG at their cell surface may explain these differences [19]. Indeed, and although not experimentally validated, it could be proposed that long-lived plasma cells producing anti-SSA/SSB Abs [20,21] are unaffected by imlifidase in our patient, allowing the recovery of auto-Abs to the initial values, while anti-HLA and particularly DSA specific memory B cells are affected by the capacity of imlifidase to cleave IgG-type of B cell receptor, thus preventing memory B cell activation and in turn Ab rebound [9].

Such an observation, better comprehension of DSA producing memory B cells, and DSA rebound may have implications in future management of highly sensitized recipients as well as in ABO-incompatible transplants. The existence of two different groups in Abs recovery is reinforced by follow-up of anti-GBM Abs coupled or not with anti-neutrophil cytoplasmic Abs (ANCA) after imlifidase injection in patients with Goodpasture disease and severe kidney injury. Indeed, in an open-label phase 2a study [14] and in the GOOD-IDES-01 trial [22] using imlifidase but not IVIg nor rituximab add-on, two-thirds of the patients underwent anti-GBM Abs recovery in the six months following imlifidase injection, and one-third did not.

In conclusion, imlifidase treatment was well tolerated in this case report, and converted a positive crossmatch to negative at H6. The results further demonstrated at the depletion phase that desensitization with imlifidase is independent from Ab specificity, while at Ab recovery/rebound phase differences may be observed as reported between auto- and allo-reactive Abs. This opens new perspectives for preventing humoral rejection. Such an effect is particularly true for anti-HLA Abs rebound that was minor at D4, and with all DSA remaining under the positivity threshold from H6 to M6. Due to the heterogeneity observed between Abs specificity, and between patients, a careful and intensive follow-up including at long-term is strongly suggested for these patients.

## Funding

This research did not receive any specific grant from funding agencies in the public, commercial, or not-for-profit sectors.

## Ethics

Consent to conduct the study and to allow publication was received for the case, and the study was conducted in accordance with the Declaration of Helsinki Principles.

## Research support

This research received no external financial or non-financial support.

## Relationships

There are no additional relationships to disclose.

## Patents and intellectual property

There are no patents to disclose.

## Other activities

There are no additional activities to disclose.

## Declaration of competing interest

The authors declare that they have no known competing financial interests or personal relationships that could have appeared to influence the work reported in this paper.

## Data Availability

Data will be made available on request.
